# Serum from Patients with Antiphospholipid Syndrome Differentially Affects Venous and Microcirculatory Endothelial Cells

**DOI:** 10.3390/biology15171460

**Published:** 2026-08-27

**Authors:** Daniele Mancardi, Francesco Maximillian Anthony Shelton Agar, Pasquale Pagliaro, Clara Marinotti, Elisa Arrigo, Alice Barinotti, Irene Cecchi, Massimo Radin, Silvia Grazietta Foddai, Dario Roccatello, Savino Sciascia

**Affiliations:** 1 Department of Clinical and Biological Sciences, University of Torino, 10043 Torino, Italy; pasquale.pagliaro@unito.it (P.P.);; 2 Nephrology and Dialysis Unit and Center of Immuno-Rheumatology and Rare Diseases (CMID), University Center of Excellence on Nephrologic, Rheumatologic and Rare Diseases (ERK-Net, ERN-Reconnect and RITA-ERN Member), Coordinating Center of the Interregional Network for Rare Diseases of Piedmont and Aosta Valley, San Giovanni Bosco Hub Hospital, 10154 Torino, Italy

**Keywords:** anti-phospholipid, endothelial dysfunction, venous, microcirculation

## Abstract

Antiphospholipid syndrome is a systemic autoimmune disease characterized by elevated circulating antiphospholipid antibodies and a predisposition to recurrent venous and arterial thrombosis. The underlying pathophysiology of antiphospholipid syndrome remains under active investigation, with endothelial dysfunction recognized as a key contributor to vascular complications. In this study, we showed that serum from antiphospholipid syndrome patients differentially affects distinct endothelial cell types, leading to variations in migration and angiogenic behavior. Using human umbilical vein endothelial cells and human microvascular endothelial cells, we assessed the biological response to serum exposure. Treatment with antiphospholipid syndrome serum was associated with an enhanced angiogenic phenotype in venous endothelium, with a significant increase in two of the four tubulogenesis parameters assessed and a trend towards increased migration, while microvascular endothelium remained largely unaffected. Consistently, metalloproteinase-9 activity was upregulated, with these effects being more pronounced in venous compared to microvascular endothelium. Moreover, caspase-3 activity remained unchanged, suggesting that caspase-3-dependent apoptosis is unlikely to be a major contributor to the endothelial response observed under these conditions. These results enhance our understanding of the pathogenesis and highlight the distinct endothelial responses to the antiphospholipid environment. Further investigation into the underlying mechanisms may facilitate the development of targeted therapeutic strategies to mitigate vascular complications.

## Simple Summary

Antiphospholipid syndrome is a systemic autoimmune disorder closely linked to the development of thrombotic events and obstetric morbidities. Although its exact pathophysiology is still under investigation, endothelial dysfunction is known to play a crucial role in vascular complications. Our work aimed to investigate the effect of APS serum collected from affected patients on endothelial cells representing the venous and microvascular districts, to evaluate potential differences in the responses of different vascular districts to the autoimmune environment. Venous and microvascular endothelial cells displayed a differential response to APS serum exposure, suggesting that the vascular complications observed in patients may derive from vascular district-specific endothelial alterations rather than from a uniform endothelial response. These findings may help clarify why antiphospholipid syndrome can affect different vascular territories and support further investigation into the mechanisms driving endothelial dysfunction in this disease.

## 1. Introduction

Antiphospholipid syndrome (APS), also known as Hughes syndrome, is a systemic autoimmune disease characterized by the presence of antiphospholipid antibodies (aPL) [1]. Most frequently, lupus anticoagulant (LA) positivity as well as anti-cardiolipin and anti-beta 2 glycoprotein I (β2GPI) antibodies have been associated with thrombotic events in venous, arterial and microcirculatory vascular beds [2]. Some studies show that aPL interfere with several targets, including soluble plasma and membrane proteins of vascular cells [3]. In turn, the interaction triggers specific signaling cascades in different cell types such as platelets, monocytes, endothelial cells, and trophoblasts. Consequently, basic cellular functions are altered, leading to an inflammatory response and thrombus formation with significant obstetric complications [4].

Nevertheless, it is crucial to consider that the activity of aPL does not directly trigger thrombotic events. Indeed, APS is characterized by the two-hit theory, in which the activity of aPL on endothelial cells would induce the development of a pro-coagulant phenotype through the previously discussed molecular mechanisms. Only after a second hit, such as vascular injury, would the thrombotic event be triggered [5].

Although increasing clinical evidence points to abnormal placental development and a high incidence of thrombotic complications in APS, the precise cellular mechanisms underlying these manifestations remain poorly understood [6]. Nevertheless, obstetric manifestations and thrombotic events have often been associated with interference with hemostasis triggered by endothelial dysfunction and complement activation [7]. APS-related thrombosis can potentially affect arteries, veins and microcirculation, leading to a broad spectrum of clinical phenotypes [8]. The heterogeneity of vascular manifestations in APS can be explained by the differential effect of aPL on endothelial cells (ECs), leading to selective endothelial dysfunction in different vascular beds [9].

It is also noteworthy that aPL development can occur in other autoimmune disorders, such as systemic lupus erythematosus (SLE). Indeed, 20 to 40% of SLE patients are aPL-positive. Moreover, about 50 to 70% of aPL-positive SLE patients develop clinical features of APS within 20 years of follow-up [10].

Considering the central role of the endothelium in the development of thrombi [11], the role of different types of ECs deserves specific attention to develop targets for precision medicine approaches in treating the vascular aspect of APS.

Several studies have shown that aPL affect EC function by promoting a proinflammatory and procoagulant phenotype. IgG isolated from APS patients activate ECs both in vitro and in vivo, leading to increased expression of adhesion molecules and tissue factor [12]. In addition, aPL have been associated with increased oxidative stress and reactive oxygen species (ROS) production in endothelial cells [13]. Mitochondrial dysfunction has also been implicated in APS-related endothelial injury, as APS-derived IgG induce mitochondrial hyperpolarization and ROS generation in HUVECs [14]. Furthermore, sera from different APS patients have been shown to exert heterogeneous effects on EC activation and dysfunction, suggesting that the endothelial response may vary according to both the characteristics of the autoimmune milieu and the EC type [15]. To the best of our knowledge, however, no study has compared the effect of APS serum on in vitro models of ECs from different vascular districts [16].

Taken together, these findings indicate that aPL influence EC function through multiple mechanisms, including inflammatory activation, oxidative stress, and mitochondrial dysfunction. However, whether ECs from different vascular districts respond similarly to APS serum remains poorly understood. Therefore, we hypothesized that APS serum may differentially affect key EC functions, including cell viability, angiogenesis, ROS production, mitochondrial function, and MMP-9 activity.

To address this hypothesis, we compared human umbilical vein endothelial cells (HUVECs), as a model of venous endothelium, and human microvascular endothelial cells-1 (HMEC-1), as a model of microvascular endothelium. This approach allowed us to evaluate whether APS serum induces distinct functional responses in different endothelial compartments.

## 2. Materials and Methods

This work was designed to specifically elucidate new putative endothelial mechanisms which are activated by the exposure to APS serum and how they are related to specific function of microvascular ECs compared to venous. To address this biological scenario, we evaluated the in vitro response of human endothelial cells obtained from the umbilical vein and from microcirculation to the administration of serum from APS patients. Hereafter, this whole patient serum will be referred to as “APS serum”, in order to distinguish it from purified aPL. Specifically, this study compared the effects of APS serum and healthy control serum on venous (HUVEC) and microvascular (HMEC-1) ECs by evaluating viability, migration, angiogenesis, apoptosis, oxidative stress, mitochondrial function, and MMP-9 activity.

Clinical characteristics of recruited patients are listed in Table 1, and antibody titers and comorbidities for each patient in Table 2. Given that patients were not all evaluated through the same laboratory assay, absolute titers were not directly comparable. Therefore, antibody levels are presented as relative categories (low, medium, high). All patients provided written informed consent, and the study was approved by the Interhospital Ethics Committee (approval #00323/2020) in accordance with the Declaration of Helsinki.

**Cell Culture**

Human umbilical vein endothelial cells (HUVEC, C-12203, PromoCell GmbH, Heidelberg, Germany) and human microvascular endothelial cells (HMEC-1, CRL-3243, ATCC, Manassas, VA, USA) were cultured in EndoGRO-MV Complete Culture Media Kit (SCME004, Merck-Millipore, Billerica, MA, USA), with Pen/Strep (P0781, Sigma-Aldrich, St. Louis, MO, USA) (1% *v*/*v*) and incubated at 37 °C, 5% CO_2_, in a HF151 CO_2_ incubator (Heal Force, Shanghai, China). Cells were periodically split when 80–90% confluence was reached. HUVEC are primary cells, whereas HMEC-1 is an immortalized line; both were used at passage 5. Seeding densities were selected for each assay format and endpoint in order to reach the confluence required by the specific readout. A confluent monolayer was necessary for the scratch-migration assay, whereas sub-confluent cultures were required for the other assays. On a per-surface-area basis, the densities used were comparable across assays. For treatment, EndoGRO-MV (FBS 0% *v*/*v*, Pen/Strep 1% *v*/*v*) was supplemented with APS patients’ serum, and a commercially available human serum derived from a pool of healthy donors (HC, H4522, Sigma-Aldrich, St. Louis, MO, USA) was used as negative control. A serum obtained from a large pool of healthy donors was selected as the reference condition, as it provides a standardized baseline, consistent between batches, and representative of the average healthy population. This choice minimizes the inter-individual and technical variability of individually collected control sera, therefore isolating the APS-specific response against a stable reference.

**Cell Viability Assay**

HUVEC and HMEC-1 cells were seeded on 96-well plates (CC7682-7596, STARLAB International GmbH, Hamburg, Germany) (1 × 10^4^ cells/well) in complete EndoGRO-MV medium and incubated overnight for adhesion at 37 °C. The following day, cells were treated with regular medium (FBS 0% *v*/*v*) supplemented with APS or control serum (HC) for 24 h. Three different concentrations of APS serum were tested (1%, 5%, 10%) to evaluate potential dose-response effects. Following treatment, MTT (M5655, Sigma-Aldrich, St. Louis, MO, USA) solution was prepared by dissolving 3-(4,5-dimethyl-2-thiazolyl)-2,5-diphenyl-2H-tetrazolium bromide in Dulbecco’s phosphate-buffered saline (DPBS) (D8537, Sigma-Aldrich, St. Louis, MO, USA) to obtain a 5 mg/mL concentration. Thus, the serum-supplemented medium was replaced with regular medium (FBS 0% *v*/*v*), and 10 µL of MTT solution was added in each well. After 3 h, absorbance was measured at 590 nm through an EnSpire^®^ Multimode Plate Reader (PerkinElmer, Waltham, MA, USA). A total of 16 wells were analyzed for each condition. For each serum concentration, 4 patients’ sera were evaluated.

**Cell Migration Assay**

Cells (4 × 10^5^) were seeded on 12-well plates (353043, Corning Incorporated, Corning, NY, USA) fitted with a 3-chamber silicone insert (80369, ibidi GmbH, Gräfelfing, Germany) and incubated overnight at 37 °C (5% CO_2_). The next day the insert was removed to create gaps in the cell monolayer, and cultures were rinsed with sterile PBS (D8662, Sigma-Aldrich, St. Louis, MO, USA) (with calcium and magnesium). Experimental groups were treated with APS patients’ serum (10% *v*/*v*) and the control group with human serum (HC, 10% *v*/*v*). The same procedure was carried out on both HUVEC and HMEC-1 cell lines. Microscopy images were acquired on an Axiovert200 microscope (Carl Zeiss, Göttingen, Germany) (magnification 5×) at time 0 (t0) and after six hours (t6). A minimum of five regions of interest were sampled for each well, and for each condition at least 4 replicates were analyzed. A total of 7 patients’ sera were tested. Images were analyzed with Fiji software (version 2.16.0).

**Tubulogenic Assay**

Tubulogenic potential was evaluated through Corning^®^ Matrigel^®^ matrix (356237, Corning Incorporated, Bedford, MA, USA); HUVEC and HMEC-1 were cultured on 24-well plates (353047, Corning Incorporated, Corning, NY, USA) and incubated at 37 °C, 5% CO_2_. After 16 h of treatment (see above), tubule formation was assessed with an Axiovert200 microscope (5× lens). Images were acquired and analyzed with Infinity software (Lumenera Corporation, Ottawa, ON, Canada) and the analysis was performed with the Angiogenesis Analyzer (version 1.0.c) plug-in by Fiji. The following parameters were considered for data analysis: number of master segments, number of master junctions, total length of master segments, and number of isolated segments. For each condition, a minimum of 4 replicates were evaluated, and 6 patients’ sera were analyzed.

**Caspase-3 Activation Assay**

To evaluate the effect of APS serum on ECs apoptotic process, caspase-3 activation was measured through a fluorescence-based assay kit (Caspase-3 Activation Assay Kit, CASP3F, Sigma-Aldrich, St. Louis, MO, USA). Initially, cells were seeded on black 96-well plates (137101, Thermo Fisher Scientific, Waltham, MA, USA) (2 × 10^4^ cells/well) in complete EndoGRO-MV medium and incubated overnight to allow adhesion at 37 °C, 5% CO_2_. The following day, 24 h treatment with 1 µM doxorubicin hydrochloride (PHR1789, Sigma-Aldrich, St. Louis, MO, USA) was performed on positive controls. At 6 h prior to the end of doxorubicin treatment, the remaining wells were exposed to serum-free medium supplemented with control serum (HC, 10% *v*/*v*) or APS serum (10% *v*/*v*), for 6 h. Following treatment, the assay was performed as per the manufacturer’s instructions, and for each condition, 3 replicates were analyzed. For this assay, 3 patients’ sera were evaluated.

**DCF-DA Assay**

ROS production under APS serum exposure was evaluated through a dichlorofluorescein diacetate (DCF-DA) assay. Cells (HUVEC and HMEC-1) were seeded in 24-well plates (2 × 10^4^ cells/well) and incubated overnight at 37 °C, 5% CO_2_. The following day, treatment with regular medium (0% FBS) supplemented with APS serum (10% *v*/*v*) or control serum (HC, 10% *v*/*v*) was performed for 24 h. After treatment, 2′,7′-dichlorofluorescein diacetate (D6883, Sigma-Aldrich, St. Louis, MO, USA) was dissolved in DMSO (445103, Carlo Erba Reagents, Cornaredo, Milan, Italy) to obtain a 10 mM solution, which was subsequently diluted in PBS to reach a 10 µM solution. Cells were rinsed twice with PBS with calcium and magnesium, and 300 µL of DCF-DA solution was added in each well. Afterwards, the cells were incubated at 37 °C, 5% CO_2_ for 45 min. Finally, the cells were rinsed twice in PBS with calcium and magnesium. Images were acquired through an Axiovert200 microscope. A total of 8 patients’ sera were tested, and for each condition a minimum of 4 replicates were analyzed. Image analysis was performed through Fiji software.

**JC-1 Mitochondrial Membrane Potential Assay**

HUVEC and HMEC-1 cells were seeded in black 96-well plates at a density of 1 × 10^4^ cells/well in complete EndoGRO-MV medium and allowed to adhere overnight at 37 °C. The following day, cells were treated for 24 h with serum-free regular medium (0% FBS, *v*/*v*) supplemented with APS serum (10% *v*/*v*) or control serum (HC, 10% *v*/*v*). Subsequently, mitochondrial membrane potential was assessed using a JC-1 Mitochondrial Membrane Potential Assay Kit (HY-K0601, MedChemExpress LLC, Monmouth Junction, NJ, USA). For depolarization controls, CCCP was added 10 min prior to the end of the treatment at a final concentration of 50 µM. After the treatment period, the medium was replaced with JC-1 working solution (2 µM in PBS), and cells were incubated for 30 min at 37 °C. Wells were then washed twice with HBSS (HY-K3034, MedChemExpress LLC, Monmouth Junction, NJ, USA), and fluorescence was sequentially recorded at 590 nm (aggregates) and 527 nm (monomers) through an EnSpire^®^ Multimode Plate Reader. Mitochondrial membrane potential was assessed by calculating the red/green fluorescence ratio (590/527 nm). Since this readout is expressed as a ratio between aggregate and monomer fluorescence, it is intrinsically normalized to mitochondrial content and cell number. Moreover, cells were seeded at a fixed density in all conditions. A minimum of 4 wells were evaluated for each condition, and 3 patients’ sera were evaluated.

**Zymography**

Activity of MMP-9 was quantified by zymography. At the end of the treatment, supernatant was sampled and centrifuged in a 5415 R refrigerated centrifuge (Eppendorf AG, Hamburg, Germany) (13,000 rpm for 5 min at 4 °C), and native proteins were quantified with the addition of a non-reducing Laemmli buffer (1610747, Bio-Rad Laboratories, Hercules, CA, USA) and separated through electrophoresis on tris/glycine SDS-polyacrylamide gel (Tris base: T1503, Sigma-Aldrich, St. Louis, MO, USA; Glycine: G8898, Sigma-Aldrich, St. Louis, MO, USA; SDS: L3771, Sigma-Aldrich, St. Louis, MO, USA; 30% Acrylamide/Bis solution: 1610158, Bio-Rad Laboratories, Hercules, CA, USA) (10%) with 1 mg/mL of gelatin (214340, Becton, Dickinson and Company (BD Difco), Sparks, MD, USA). Gels were then regenerated with a washing buffer (Triton X-100 (T8787, Sigma-Aldrich, St. Louis, MO, USA), 2.5% *v*/*v*; Tris-HCl (T3253, Sigma-Aldrich, St. Louis, MO, USA) (pH 7.5) 50 mM; ddH_2_O) for 1 h. Gels were thereafter kept in an incubation solution (Tris-HCl (pH 7) 40 mM; CaCl_2_ (C1016, Sigma-Aldrich, St. Louis, MO, USA) 10 mM; ddH_2_O) at 37 °C overnight. After incubation, gels were stained with 0.05% *w*/*v* Coomassie brilliant blue R-250 (1.12553, Merck, Darmstadt, Germany) for 3 h at room temperature and then destained (methanol (320390, Sigma-Aldrich, St. Louis, MO, USA) 30% *v*/*v*; acetic acid (A6283, Sigma-Aldrich, St. Louis, MO, USA) 10% *v*/*v*; ddH_2_O) for 1 h. MMP-9 activity was detected as bright bands on the blue background of gels and discoloration was proportional to activity. Each sample was evaluated in 6 independent experiments, and in total 7 patients’ sera were used as treatment.

**Statistical Analysis**

Data are presented as violin plots, with each dot representing an individual value. Each serum was tested in at least three technical replicates. Technical replicates were averaged per serum, and the individual serum donor was considered the unit of analysis for statistical evaluation. At least three independent APS sera were included in each experiment, whereas the commercially available human control serum (HC), derived from a pool of healthy donors, was treated as a single biological replicate and tested in multiple samples. One-way ANOVA or Welch’s *t*-tests were performed to evaluate statistical significance among groups, where appropriate. Specifically, APS per-serum means were compared to the pooled HC reference through a one-sample *t*-test, whereas primary and secondary APS were compared through a two-sample Welch’s *t*-test. The total number of replicates for each condition is reported in the Figures’ captions. Statistical significance refers to *p*-value as follows: * *p* < 0.05, ** *p* < 0.01, *** *p* < 0.001, **** *p* < 0.0001. Data processing, analysis, and statistics were performed with GraphPad Prism 9 (GraphPad Software, San Diego, CA, USA).

## 3. Results

### 3.1. APS Serum Exposure Did Not Significantly Alter HUVEC Viability and Tended to Reduce HMEC-1 Viability

**Figure 1:**
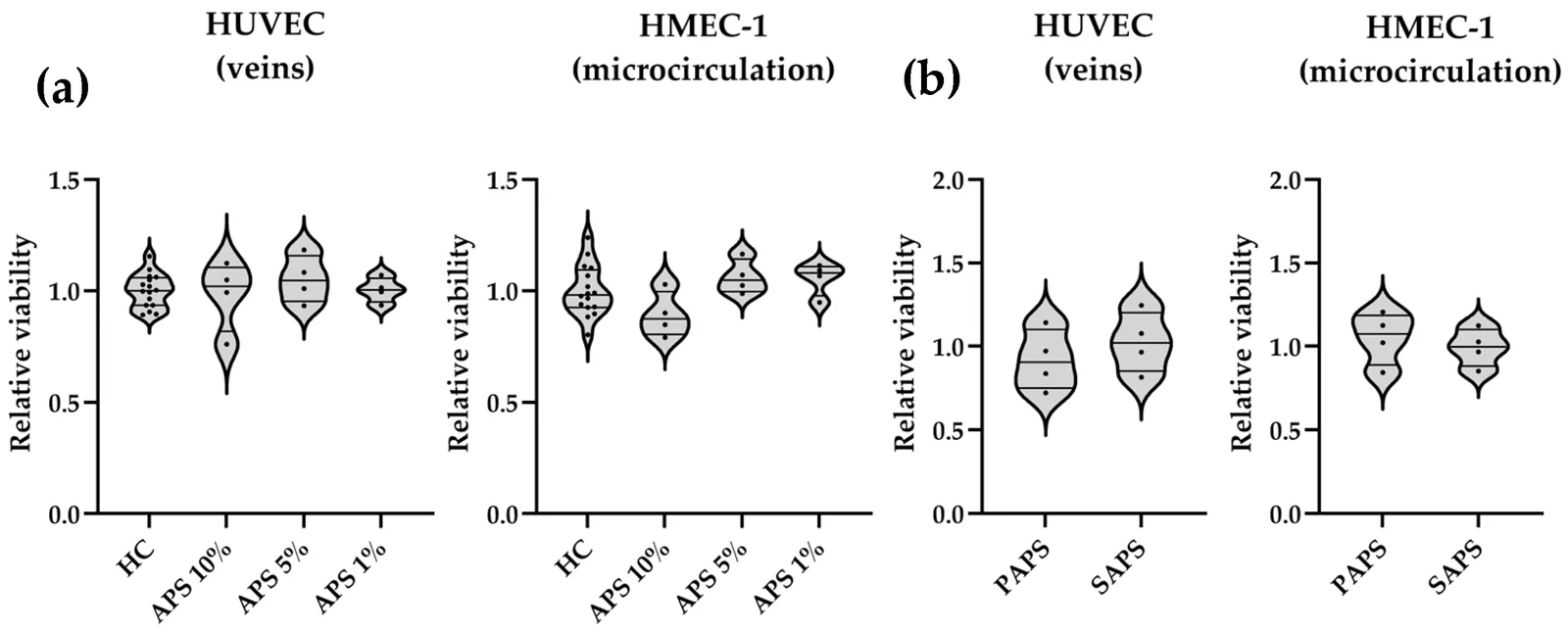
Effect of APS serum exposure on HUVEC and HMEC-1 cells viability. (**a**) Viability was addressed through MTT assay. Graphs represent HUVEC and HMEC-1 response to APS (10% *v*/*v*) or control (10% *v*/*v*) serum for 24 h. Data are presented as violin plots, with each dot representing an individual value. Biological replicates: APS, *n* = 4 sera; HC, *n* = 1 (commercial pooled serum). Technical replicates: 4 per serum for APS (total = 16 wells) and 16 for HC, compared to control (HC). (**b**) Evaluation of the differential effect of serum obtained from primary and secondary APS patients on HUVEC and HMEC-1 cells. Data are presented as violin plots, with each dot representing an individual value. Biological replicates: PAPS, *n* = 4 sera; SAPS, *n* = 4 sera. Technical replicates: 4 per serum (total = 16 each).

As shown in Figure 1a, we observed no noticeable effects on HUVEC viability following treatment with different APS serum concentrations. However, HMEC-1 viability showed a reduction after exposure to 10% APS serum for 24 h. At the serum-donor level, this reduction did not reach statistical significance (fold ≈ 0.89) and should therefore be interpreted as a trend. Given that approximately 40% of the APS cohort included patients with secondary APS (SAPS), we hypothesized a potential effect of the underlying autoimmune background on the observed results. To address this concern, an additional assay was performed to evaluate potential disparities between the effects of serum obtained from patients with primary APS (PAPS) and serum obtained from patients with secondary APS (SAPS). As shown in Figure 1b, no statistically significant differences were observed between PAPS and SAPS patients’ serum.

### 3.2. APS Serum Tended to Increase Migration in HUVEC

**Figure 2:**
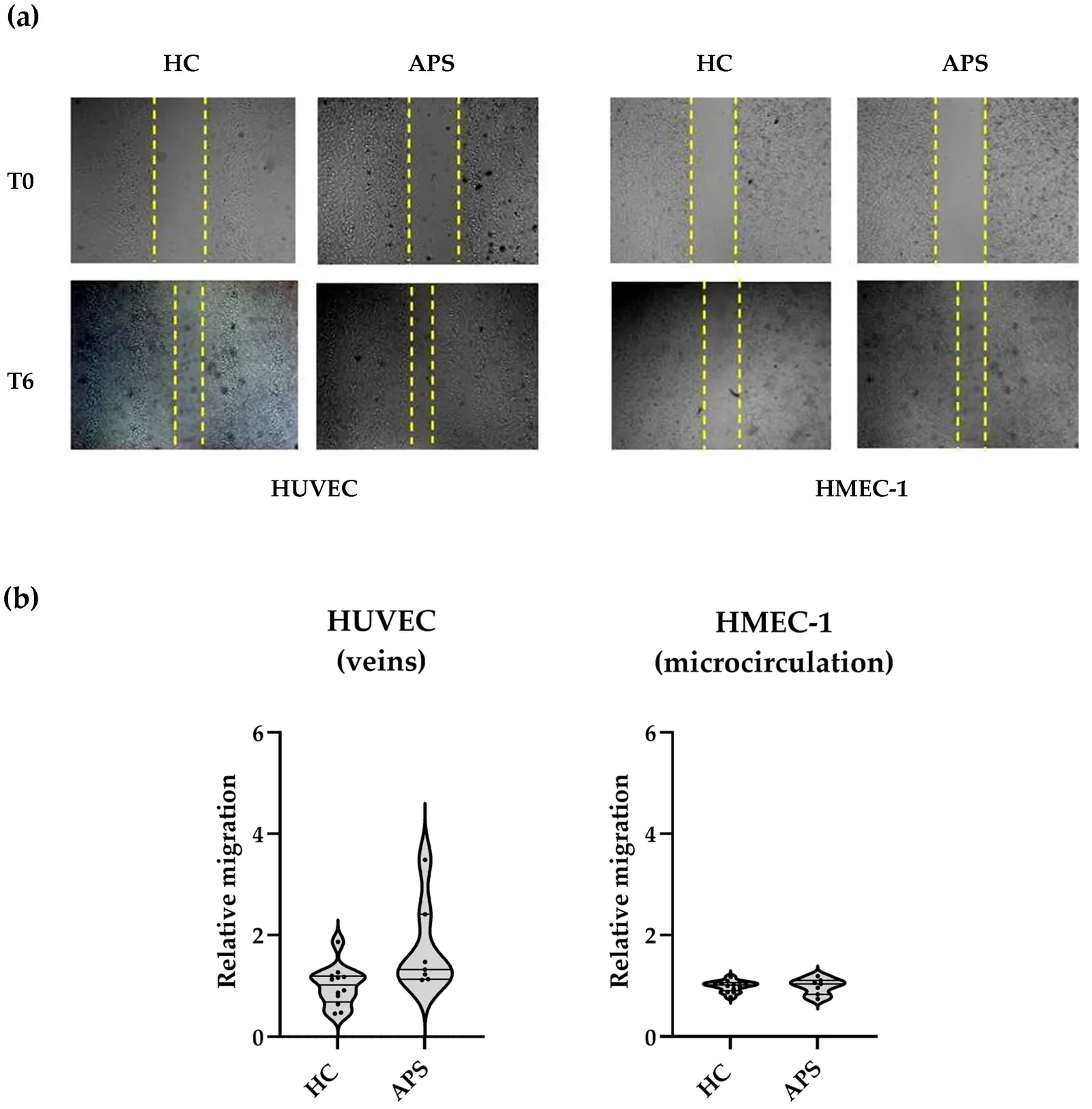
Effect of APS serum exposure on HUVEC and HMEC-1 migration. (**a**) Representative images of specimens during the treatment. Photos were acquired using an Axiovert200 microscope at 5× magnification at the beginning of treatment (T0) and after 6 h (T6); The dotted lines represent the wound front at T0; the area between the lines corresponds to the gap progressively closed by migrating cells. (**b**) Graph represents HUVEC and HMEC-1 cells response to APS (10% *v*/*v*) or control (10% *v*/*v*) serum treatment for 6 h. Data are presented as violin plots, with each dot representing an individual value. Biological replicates: APS, *n* = 7 sera; HC, *n* = 1 (pooled serum). Technical replicates: 4 per serum for APS (total = 28) and 12 for HC.

Migration capacity is a crucial feature for ECs representing a fundamental step in angiogenesis and wound healing processes, among others [17]. Our data show that APS serum increased HUVEC migration compared to control serum (Figure 2b). At the serum-donor level, this increase represented a strong trend (≈73% increase), whereas in HMEC-1 no differences could be observed compared to the control group. This result may suggest a heightened effect of APS serum on venous endothelium, compared to microcirculation.

### 3.3. APS Serum Treatment Enhanced Tubulogenesis in HUVEC

Angiogenesis can be both pathological and physiological as in many autoimmune diseases or in chronic inflammation [18]. Migration and viability results suggest a putative role for tubulogenesis in the development of vascular events induced by APS serum. Therefore, HUVEC and HMEC-1 were tested for the formation of tubule-like structures under control (HC, 10% *v*/*v*) and APS (10% *v*/*v*) serum treatment for 6 h.

**Figure 3:**
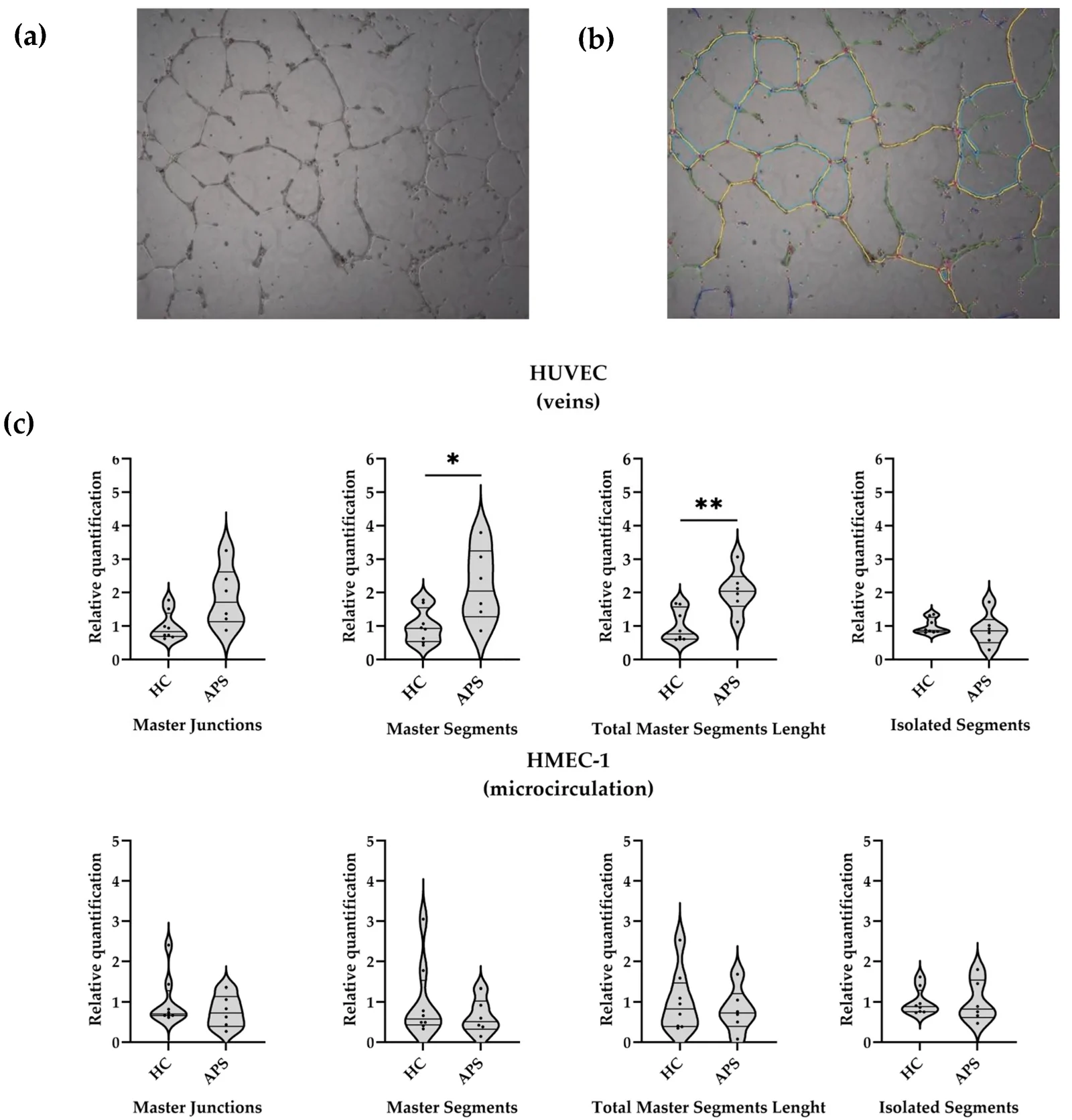
Effect of APS serum on HUVEC and HMEC-1 cells angiogenesis behavior. (**a**) Representative image acquired through an Axiovert200 microscope at 5× magnification, showing tubule formation in human umbilical vein endothelial cells (HUVEC). (**b**) Same image after software processing through the Fiji plugin Angiogenesis Analyzer. (**c**) Graphs represent the relative quantification of master junctions, master segments, total master segments’ length, and isolated segments produced by HUVEC and HMEC-1 cells following APS (10% *v*/*v*) or control (10% *v*/*v*) serum exposure for 6 h. Data are presented as violin plots, with each dot representing an individual value. Biological replicates: APS, *n* = 6 sera; HC, *n* = 1 (pooled serum). Technical replicates: 4 per serum for APS (total = 24) and 8 for HC. *p*-values: * *p* < 0.05, ** *p* < 0.01, compared to control.

As shown in Figure 3c, capillary-like structures were more abundant in APS treated HUVEC compared to control, with an increased number (2.2-fold) and length (2.05-fold) of master segments, as well as an increased number of master junctions (1.86-fold, trend). Conversely, no significant difference was observed in the number of isolated segments (Figure 3c). This pattern is reversed in HMEC-1, which showed a trend towards a reduced formation of capillary-like structures (Figure 3c), matching the poor results in terms of migration (Figure 2b). Our results suggest an increased angiogenic activity in the venous endothelium in response to APS serum, as opposed to microcirculation.

### 3.4. APS Serum Failed to Activate Caspase-3 in HUVEC and HMEC-1

Internal or external stimuli can lead to the initiation of the apoptotic process, which can develop through an extrinsic pathway or an intrinsic, mitochondrial pathway. Both pathways lead to the activation of a cascade of proteases known as caspases, which are synthesized as proenzymes and activated following proteolytic cleavage. Their active form may then cleave and activate other caspases.

Given that both pathways lead to the activation of caspase-3, it is considered the main effector caspase responsible for the apoptotic process [19]. Therefore, evaluating its activity can be an effective method to determine the induction of apoptosis.

**Figure 4:**
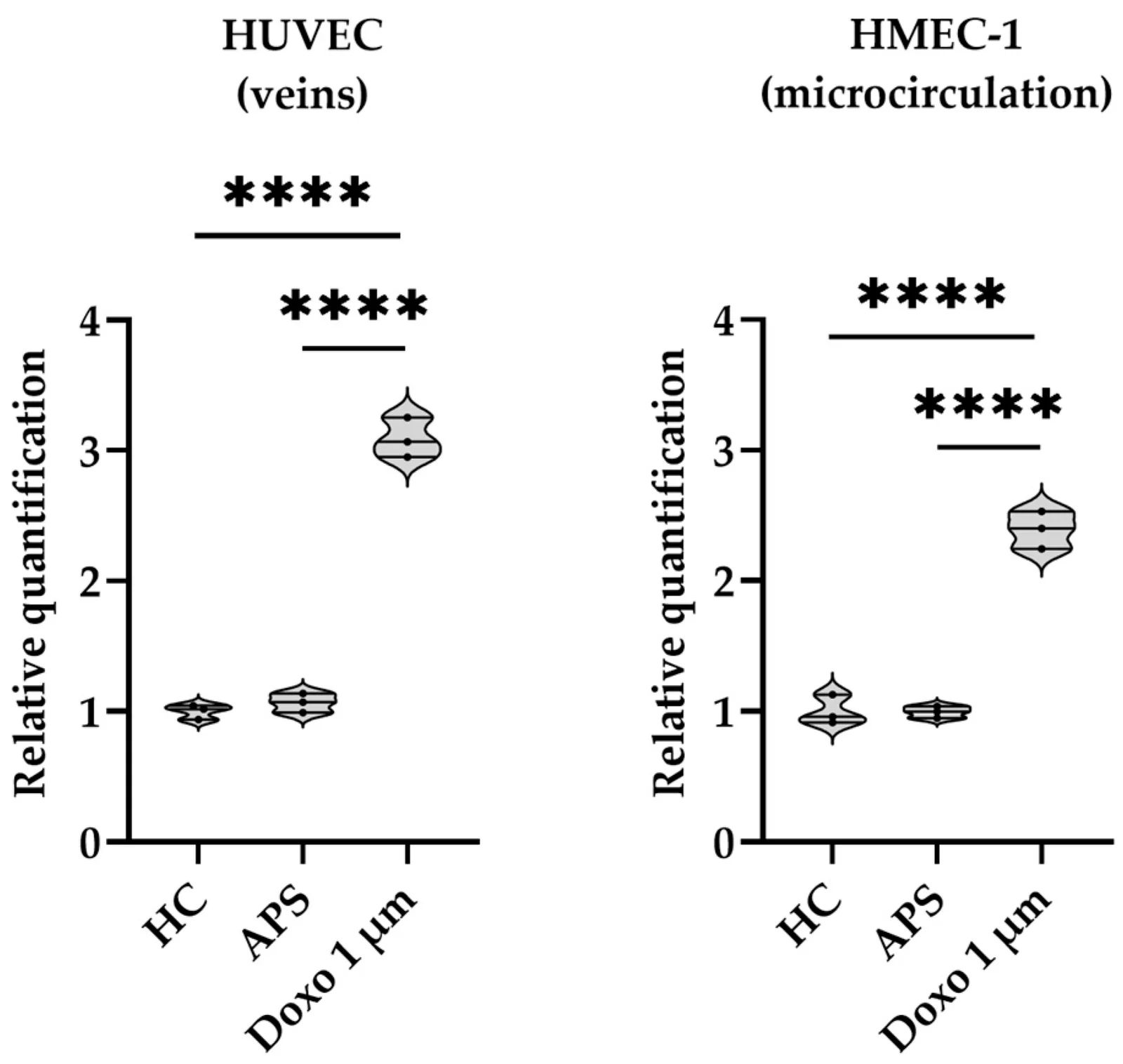
Effect of APS serum exposure on caspase-3 activity. Graphs represent the relative quantification of the fluorescence signal intensity produced by the caspase-3 substrate in HUVEC and HMEC-1 cells following APS (10% *v*/*v*) or control (10% *v*/*v*) serum exposure for 6 h. Exposure of 24 h to 1 µM doxorubicin (Dox) was used as a positive control condition. Data are presented as violin plots, with each dot representing an individual value. Biological replicates: APS, *n* = 3 sera; HC, *n* = 1 (pooled serum). Technical replicates: 3–5 per condition (per serum for APS); doxorubicin (Dox) served as the positive control. *p*-value: **** *p* < 0.0001.

As shown in Figure 4, treatment with APS serum failed to activate caspase-3 in both HUVEC and HMEC-1 cells, as no difference was observed between control (HC) and treated (APS) groups, whereas cells treated with 1 µM doxorubicin hydrochloride exhibited an enhanced caspase-3 activity.

### 3.5. APS Serum Exposure Enhanced the Generation of Reactive Oxygen Species

The generation of reactive oxygen species can occur in both physiological and pathological conditions. Indeed, they play a crucial role in proper intracellular signaling, functioning as second messengers and regulating various processes through proliferative, apoptotic, fibrotic, or inflammatory signals. However, excessive ROS production is linked to pathological conditions [20]. Therefore, to investigate a potential alteration in ROS production following APS serum exposure, a DCF-DA assay was performed.

**Figure 5:**
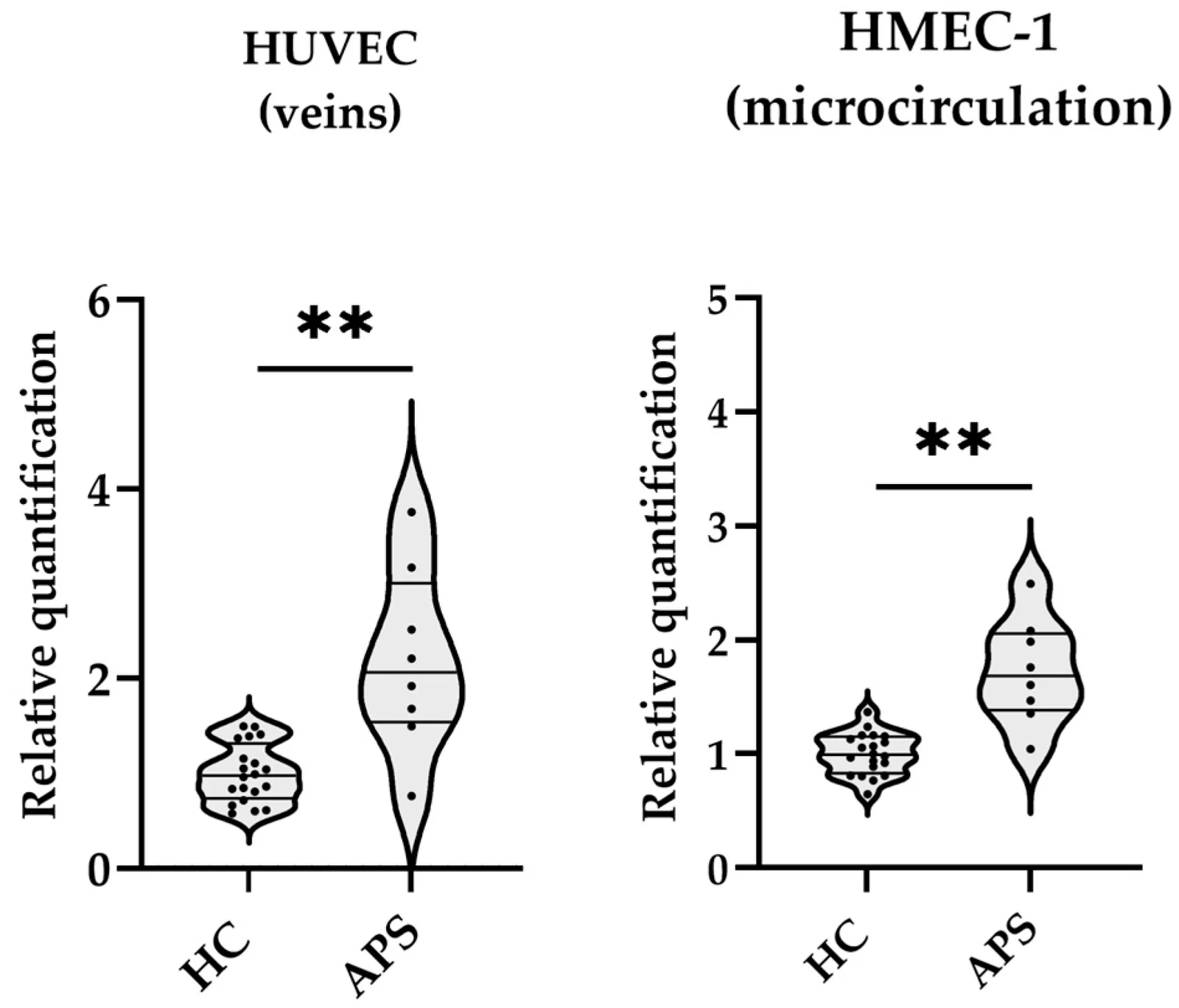
Effect of APS serum exposure on HUVEC and HMEC-1 ROS production. Production of reactive oxygen species was evaluated through a DCF-DA assay. Graphs represent HUVEC and HMEC-1 response to APS (10% *v*/*v*) or control (10% *v*/*v*) serum for 6 h. Data are presented as violin plots, with each dot representing an individual value. Biological replicates: APS, *n* = 8 sera; HC, *n* = 1 (pooled serum). Technical replicates: 20 per serum for APS (total = 160) and 20 for HC. *p*-value: ** *p* < 0.01, compared to control (HC).

Cellular fluorescence intensity was quantified as it is proportional to ROS production. As shown in Figure 5, treatment with APS serum resulted in an increased reactive oxygen species production both in HUVEC and HMEC-1 cells, compared to control conditions (HC) (HUVEC, 2.19-fold; HMEC-1, 1.72-fold).

### 3.6. APS Serum Exposure Tended to Increase Mitochondrial Membrane Potential in HUVEC and HMEC-1

**Figure 6:**
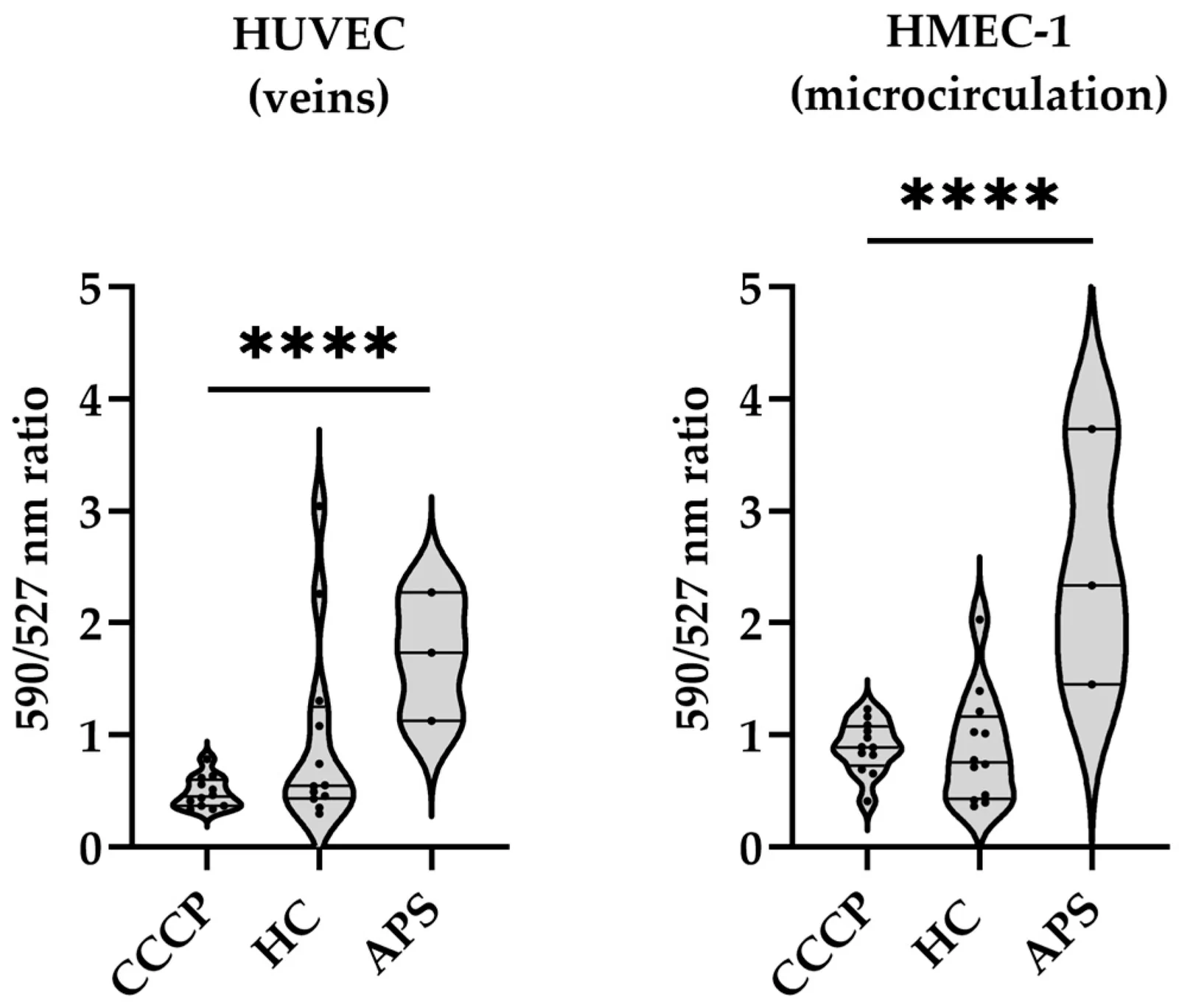
Effect of APS serum exposure on HUVEC and HMEC-1 mitochondrial membrane potential. The mitochondrial membrane potential was assessed through JC-1 staining. Graphs represent the red/green fluorescence ratio detected following APS (10% *v*/*v*) or control (10% *v*/*v*) serum treatment for 24 h. Data are presented as violin plots, with each dot representing an individual value. CCCP: *n* = 12 replicates. Biological replicates: APS, *n* = 3 sera. HC, *n* = 1 (pooled serum). Technical replicates: 4 per serum for APS (total = 12) and 12 for HC. *p*-value: **** *p* < 0.0001, compared to depolarization control (CCCP).

Mitochondrial membrane potential (ΔΨm) is a specific marker of mitochondrial function and integrity and can be altered in oxidative stress; in endothelial cells, increased ΔΨm has been associated with increased ROS production following exposure to aPL [14]. Therefore, to evaluate the effect of APS serum on mitochondrial membrane potential, HUVEC and HMEC-1 cells were stained with JC-1 dye following treatment. As shown in Figure 6, the red/green fluorescence ratio was higher in cells exposed to APS serum than in the CCCP depolarization control; at the serum-donor level, the increase over HC serum represented a non-significant trend (HUVEC, 1.78-fold; HMEC-1, 2.85-fold).

### 3.7. APS Serum Enhanced MMP-9 Activity in HMEC-1 and HUVEC

Extracellular matrix (ECM) degradation is a key interaction during angiogenesis and matrix metalloproteinase-9 (MMP-9) is often involved in pro-angiogenic processes. It is a zinc-dependent endopeptidase playing a critical role in the regulation of multiple biological processes, including ECM remodeling and degradation. MMP-9 activity is involved in various pathological conditions characterized by ECM remodeling; additionally, it has been demonstrated that MMP-9 promotes angiogenesis and cell migration in cancer cells [21].

**Figure 7:**
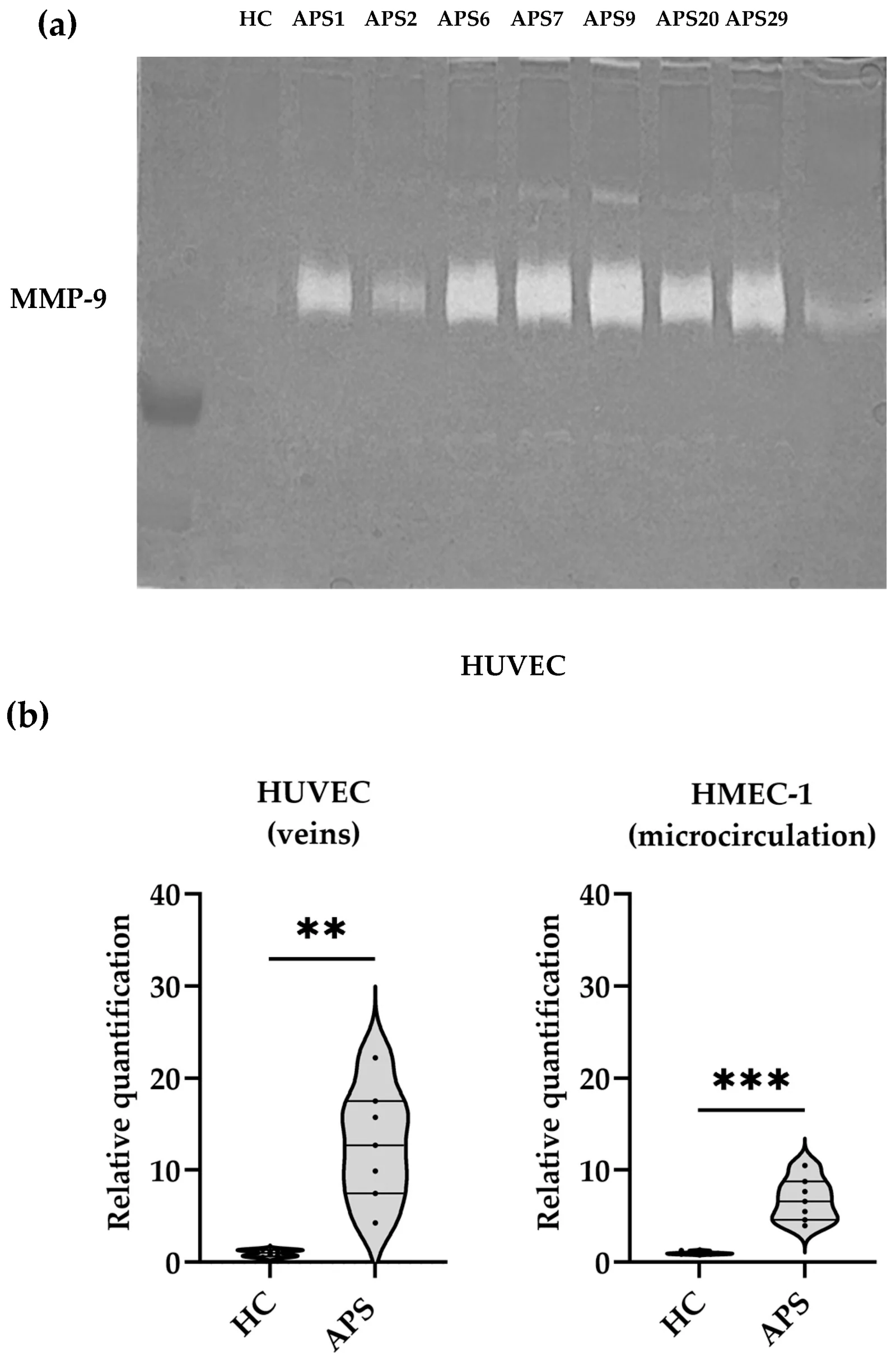
Effect of APS serum exposure on HUVEC and HMEC-1 cells’ MMP-9 activity. (**a**) Image shows a representative picture of the assay performed to measure MMP-9 activity on HUVEC through gelatin zymography; HC line is referred to as human control, whereas APS lines refer to APS serum. MMP-9 activity appears to be directly proportional to the bands’ intensity. (**b**) Graphs represent MMP-9 quantification in centrifuged media obtained from HUVEC and HMEC-1 cell cultures following APS (10% *v*/*v*) or control (10% *v*/*v*) serum exposure for 6 h. Data are presented as violin plots, with each dot representing an individual value. Biological replicates: APS, *n* = 7 sera; HC, *n* = 1 (pooled serum). Technical replicates: 6 per serum for APS (total = 42) and 6 for HC. *p*-values: ** *p* < 0.01, *** *p* < 0.001, compared to control.

Given the available literature data, we investigated MMP-9 levels in conditioned media obtained from ECs treated with APS patients’ serum or control conditions. As shown in Figure 7b, we observed an increased MMP-9 activity in cells treated with APS serum, compared to controls; this effect is particularly pronounced in HUVEC cells (12.8-fold), compared to HMEC-1 (6.8-fold), despite being elevated in both cell lines.

## 4. Discussion

Our study investigated endothelial dysfunction in APS, highlighting possible functional differences among endothelial cell lines. An experimental in vitro endothelial model was established, using primary HUVEC and HMEC-1 to investigate differences between the two lines when exposed to serum from patients with APS. Initially, APS patients’ serum was tested on ECs to assess their viability following serum exposure (Figure 1a). As a result, no significant alterations in cell viability were observed in HUVEC cells after 24 h treatment with different APS serum concentrations. On the other hand, exposure to 10% APS serum resulted in decreased viability in HMEC-1, which did not reach statistical significance at the serum-donor level, as shown in Figure 1a. This apparent decrease in viability may not fully reflect the in vivo condition, as in APS patients the endothelium is chronically exposed to circulating autoantibodies, yet cell death is not described. This discrepancy suggests a potential role of compensatory mechanisms in the development of a certain degree of tolerance towards autoantibodies, therefore preventing cell death. However, such mechanisms remain poorly characterized, and further investigations are required to gain deeper insight into this aspect.

Given that secondary APS is frequently associated with other autoimmune conditions, an additional MTT assay was performed to evaluate whether serum obtained from SAPS patients could have a differential effect on cells compared to serum obtained from PAPS patients. As shown in Figure 1b, no significant differences were observed between the two groups, suggesting that the effects observed are primarily attributable to APS rather than to concomitant autoimmune conditions.

The effect of APS serum on HUVEC and HMEC-1 angiogenic properties was also assessed, showing pro-angiogenic responses in HUVEC, as both cellular migration (Figure 2b) and capillary network formation (Figure 3) could be observed in treated cells. The same behavior was not observed in HMEC-1, suggesting a different response of the two vascular systems. We also observed an increased angiogenic activity in HUVEC as a response to APS serum exposure, demonstrated by a higher number and total length of master segments (Figure 3c). The same behavior could not be observed in HMEC-1, as shown in Figure 3c. We also noticed a trend suggesting an increasing number of isolated segments in HMEC-1 (Figure 3c). Several mechanisms may have led to the differential angiogenic activity observed in HUVEC and HMEC-1. For instance, CD93 is a receptor expressed on the surface of endothelial cells and monocytes, and it plays an important role in both angiogenesis and inflammation. Its downstream effectors include RAC1, a small GTPase involved in cell migration. Moreover, CD93 has been shown to be upregulated in various autoimmune diseases [22]. Its differential expression, following APS serum exposure, may explain the observed differences between HUVEC and HMEC-1, highlighting a possible mechanistic link between APS-related factors and endothelial dysfunction. This interpretation remains hypothetical and requires further studies to be confirmed.

Given the results obtained in functional assays, we next investigated whether APS serum could induce apoptosis in HUVEC and HMEC-1 cells. To achieve this, a Caspase-3 Activation Assay Kit was implemented to assess caspase-3 activation following treatment, as it is a key effector in both the intrinsic and extrinsic apoptotic pathways [19]. The obtained results confirmed caspase-3 activation in response to 1 µM doxorubicin exposure, which served as a positive control. No significant differences were observed comparing APS and control serum treatment (Figure 4), suggesting that APS serum does not induce caspase-3-dependent apoptosis in either cell line. This result suggests that findings obtained from the viability assays are more likely to be attributed to a mechanism possibly involving mitochondrial dysfunction rather than a cell death process. The increase in ROS in the absence of caspase-3 activation may suggest a non-apoptotic outcome, such as senescence or mitophagy. Future studies using Annexin V/PI staining, the Bax/Bcl-2 ratio and senescence markers (p16, SA-β-gal) will be required to characterize this aspect. This is consistent with the established association between oxidative stress, mitochondrial dysfunction, and the pathogenesis of APS [23]. An alternative mechanism leading to the differential results observed in viability assays and molecular analysis could be represented by a PINK1-dependent mitophagy. Indeed, mitophagy is a protective mechanism triggered by mitochondrial damage, and it is known that PINK1 plays a pivotal role in mitophagy [24]. These prior considerations led our study to direct its focus towards the analysis of ROS production in both HUVEC and HMEC-1 cells treated with APS patients’ serum, aiming to determine whether APS serum exposure could lead to increased oxidative stress. As expected, ROS production was increased in both cell lines following APS serum treatment (Figure 5). Our findings highlighted the need to investigate mitochondrial activity in greater depth; therefore, we performed JC-1 staining on HUVEC and HMEC-1 cells following 24 h treatment with APS or control serum. APS serum induced a slight increase in the red/green fluorescence ratio in both HUVEC and HMEC-1 cells, suggesting a potential mitochondrial hyperpolarization (Figure 6). Despite mitochondrial depolarization being generally associated with the initiation of apoptosis, there is growing evidence supporting mitochondrial hyperpolarization leading to a dysfunctional state. Moreover, previous studies on endothelial cells observed mitochondrial hyperpolarization following exposure to aPL, concomitant with oxidative stress [14]. Our results are consistent with these findings and support the interpretation that APS serum exposure leads to mitochondrial stress. This suggests that ΔΨm hyperpolarization may serve as an indicator of endothelial dysfunction in APS, potentially contributing to oxidative stress-driven vascular damage. However, JC-1 staining alone cannot establish mitochondrial dysfunction, and a causal role of oxidative stress cannot be inferred from these data. Further assays, including ATP production, oxygen consumption rate, mitochondrial-targeted ROS, and the kinetics of ΔΨm, will be required to confirm this interpretation. Moreover, at the serum-donor level, the JC-1 changes represented a non-significant trend.

Finally, as a pivotal step in new vessel sprouting, we investigated the activity of matrix metalloproteinases (MMPs) through gelatin zymography (Figure 7). MMP activity is essential for degrading extracellular matrix proteins, as well as for vascular tissue remodeling within processes such as angiogenesis and wound healing; moreover, their activity promotes proliferation, differentiation and migration [25]. Therefore, in our study, we assessed the activity of MMP-9 upon APS serum exposure, as there is evidence supporting its prevalent expression in endothelial cells [25]. APS serum exposure induced a significant increase in MMP-9 activity, particularly in HUVEC compared to HMEC-1 cells (Figure 7b). This observation was consistent with the results of the cell migration assay and the tubulogenic assay, particularly in HUVEC cells (Figure 2b and Figure 3c).

The obtained results indicate that HUVEC and HMEC-1 cell lines demonstrate distinct responses following exposure to APS serum. These differences may reflect a differential effect of APS serum on endothelial activation, which could be explained by the increased migration and tubule-like structure formation observed in HUVEC compared to HMEC-1, as well as the more pronounced increase in ROS production and MMP-9 activity. This interpretation remains hypothetical, and further studies will be required to confirm this potential divergence.

Indeed, the precise molecular mechanisms underlying this differential response remain incompletely elucidated and will be the subject of future investigations. For instance, the divergent response observed in terms of cell migration may depend on a differential activation of the p38-MAPK pathway, given its significant role in endothelial cell migration [26]. However, as mentioned previously, confirmation of these hypotheses will be the focus of future studies. Consequently, additional investigations are warranted to delineate the primary variances in molecular activation triggered by the APS serum treatment. An alternative explanation is that aPL interact with a different set of receptors in the two endothelial cell types. Indeed, in addition to β2GPI-dependent pathways, ApoER2 and other members of the LDL-receptor family, TLR2, TLR4, and Annexin A2 have been implicated in APS [5]. Endothelial cells obtained from different vascular beds display a well-documented intrinsic heterogeneity [27,28], and a differential expression of these receptors between venous and microvascular endothelium could therefore contribute to the divergent responses observed. Heparanase has also been implicated as a mediator of aPL-induced endothelial and platelet activation, as its inhibition reduces tissue factor expression and NF-κB activation triggered by anti-β2GPI antibodies [29]. Establishing causality will require dedicated experiments, including ROS scavengers and mitochondrial-targeted antioxidants, the assessment of NF-κB and p38-MAPK activation, the quantification of the adhesion molecules VCAM-1, ICAM-1 and E-selectin, the evaluation of candidate-receptor and heparanase expression, and the comparison between purified IgG and whole serum. These experiments are the focus of our ongoing work.

The reasons why some patients with aPL develop venous rather than arterial or microvascular events are still unclear. Nevertheless, it is crucial to further investigate the precise mechanisms involved in the development of thrombotic events, particularly those associated with venous and arterial phenomena. It has been demonstrated that aPL can induce the expression of adhesion molecules and tissue factor following endothelial activation [30]. Moreover, they can bind to surface receptors such as apolipoprotein E receptor 2, leading to the activation of the p38-MAPK and NF-κB pathways, promoting an inflammatory phenotype which may contribute to thrombosis [5]. However, further studies are necessary to identify the specific mechanisms that distinguish thrombosis at the venous or arterial levels.

Our findings might contribute to our understanding of pathogenesis of the syndrome. It is crucial, however, to highlight that further experiments will be required to gain a better understanding of the role of APS serum in endothelial activation and the extent of functional alteration occurring in the venous compartment and microcirculation. Moreover, single or double aPL-positive patients were not included in this study. However, a selective evaluation of the effect of serum obtained from single or double aPL-positive patients could provide a deeper understanding of the contribution of each specific aPL in driving the observed cellular biological responses triggered by APS serum exposure and will be the focus of future investigations. For instance, in patients, the action of aCL has been observed to induce elevated levels of nitric oxide (NO) and superoxide, leading to the generation of plasma peroxynitrite, thereby exerting potent pro-oxidative effects. Additionally, aCL seem to bind to specific plasma enzymes with antioxidant properties, such as PON1, inhibiting their activity and consequently inducing an increase in oxidative stress. This vascular elevation of reactive oxygen species (ROS) culminates in endothelial damage, inflammation, and a heightened thrombotic risk [31]. With the updated 2023 ACR/EULAR classification criteria recently released, the importance of differentiating between micro- and macrovascular involvement in APS has been further highlighted. Future exploration of the underlying mechanisms could aid in identifying novel therapeutic strategies for treating vascular events induced by aPL.

**Limitations**

This study has several limitations. First, the number of APS patients included was limited, reflecting the rarity of the disease and reducing the statistical power and generalizability of the findings. Second, the heterogeneous antibody profiles and clinical characteristics of the patients may have contributed to the variability observed in endothelial responses. Third, the sample size did not allow robust stratified analyses according to antibody profile, clinical manifestations, disease activity, or treatment. In particular, the number of patients was insufficient to assess potential associations between endothelial responses and specific vascular phenotypes. Finally, no patients with obstetric APS were included; therefore, our findings cannot be directly extrapolated to obstetric manifestations of the syndrome. Larger and clinically stratified cohorts will be required to validate and extend these observations. Our findings should moreover be regarded as preliminary and hypothesis-generating. Whole patient serum was used rather than purified IgG, and this does not allow the effects to be attributed specifically to aPL as opposed to complement or other serum components. In addition, the healthy control consisted of a single commercial pooled serum, which cannot capture the inter-individual variability of healthy subjects. HUVEC (primary, neonatal) and HMEC-1 (immortalized, adult microvascular) also differ in origin and immortalization status, and part of the differential response may therefore reflect these intrinsic differences between the two models, in addition to vascular bed identity. At present it is therefore not possible to establish which specific serum component drives the observed effects, and future studies using purified IgG fractions will be required to clarify this point. Several endpoints, including migration, mitochondrial membrane potential, and the reduction in microvascular viability, also showed consistent trends that did not reach statistical significance at the serum-donor level. Given the small number of sera, this most likely reflects the limited statistical power afforded by the cohort size rather than a true absence of effect, and these observations will require confirmation in adequately powered studies.

## 5. Conclusions

Here we showed that APS serum is associated with distinct functional responses in venous and microvascular endothelial cells. Venous endothelial cells displayed increased tubule-like network formation and MMP-9 activity, together with a trend towards enhanced migration, whereas microvascular endothelial cells showed a trend towards reduced viability and a weaker angiogenic response. Interestingly, APS serum increased ROS production and showed a non-significant trend towards mitochondrial membrane hyperpolarization in both cell types, while caspase-3 activity remained unchanged. Given the limited number of sera and the use of whole serum, these findings should be considered preliminary and associative rather than causal. The findings suggest that endothelial dysfunction in APS involves shared alterations in oxidative stress and, possibly, in mitochondrial function together with vascular bed-specific functional responses. Although the underlying molecular pathways require further investigation, our results support the concept that endothelial heterogeneity may contribute to the diverse vascular manifestations of APS.

## Figures and Tables

**Table 1:** Clinical characteristics of recruited patients with thrombotic APS. Secondary APS was associated with SLE.

	N	%
**Clinical and demographical characteristics**		
Female	7	78%
Male	2	22%
Age (median, range)	51, 32–61	
Arterial event (ischemic stroke)	2	22%
Venous event (pulmonary embolism)	3	33%
Microvascular event (TMA or skin ulcer)	4	44%
Triple aPL positive	7	78%
Double aPL positive	1	11%
Single aPL positive	1	11%
Primary APS	5	56%
Secondary APS	4	44%
Obstetric complications	0	0%
**Therapy**		
ASA	3	33%
VKA	9	100%

**Table 2:** Relative antibody titers measured in recruited patients, as well as comorbidities.

ID	Sex	Positive Antibodies and Titers	Comorbidities
1	F	LA+, aCL IgG (medium/high)	Diabetes, hypertension
2	F	LA+, aCL IgG (medium/high), aβ2GPI IgG (high)	Hypertension, hyperlipidemia, diabetes, peripheral arterial vasculopathy, unstable angina, coronary artery disease, and mild-to-moderate renal insufficiency
6	F	LA+, aCL IgG (medium/high), aβ2GPI IgG (high)	Hypertension, chronic renal insufficiency
7	M	LA+, aCL IgG (high), aβ2GPI IgG (high), aPS/PT IgG (high), aPS/PT IgM (high)	
9	F	LA+, aCL IgG (high), aβ2GPI IgG (high)	Hypertension
12	F	LA+, aCL IgG (medium/high), aβ2GPI IgG (medium/high), aPS/PT IgG (high), aPS/PT IgM (high)	Hypertension, hyperlipidemia
20	F	aCL IgG (high), aβ2GPI IgG (high), aβ2GPI IgM (low), aPS/PT IgG (high)	
29	M	aCL IgG (high), aCL IgM (medium), aβ2GPI IgG (medium/high), aPS/PT IgG (high), aPS/PT IgM (high)	
60	M	LA+, aPS/PT IgM (high)	Hypertension, hyperlipidemia

## Data Availability

The data presented in this study are available on request from the corresponding author.
